# Comparison of Commercial Genetic-Testing Services in Korea with 23andMe Service

**DOI:** 10.1155/2014/539151

**Published:** 2014-06-25

**Authors:** Sollip Kim, Ki-Won Eom, Chong-Rae Cho, Tae Hyun Um

**Affiliations:** Department of Laboratory Medicine, Ilsan Paik Hospital, Inje University College of Medicine, Joowha-ro 170, Ilsanseo-gu, Goyang, Gyeonggi 411-706, Republic of Korea

## Abstract

*Introduction*. Genetic testing services for disease prediction, drug responses, and traits are commercially available by several companies in Korea. However, there has been no evaluation study for the accuracy and usefulness of these services. We aimed to compare two genetic testing services popular in Korea with 23andMe service in the United States. *Materials and Methods*. We compared the results of two persons (one man and one woman) serviced by Hellogene Platinum (Theragen Bio Institute), DNAGPS Optimus (DNAlink), and 23andMe service. *Results*. Among 3 services, there were differences in the estimation of relative risks for the same disease. For lung cancer, the range of relative risk was from 0.9 to 2.09. These differences were thought to be due to the differences of applied single nucleotide polymorphisms (SNPs) in each service for the calculation of risk. Also, the algorithm and population database would have influence on the estimation of relative disease risks. The concordance rate of SNP calls between DNAGPS Optimus and 23andMe services was 100% (30/30). *Conclusions*. Our study showed differences in disease risk estimations among three services, although they gave good concordance rate for SNP calls. We realized that the genetic services need further evaluation and standardization, especially in disease risk estimation algorithm.

## 1. Introduction 

Genetic testing services for disease prediction, drug responses, and personal traits are becoming available by several companies and getting more popular in Korea. These genetic services were based on the results of high-tech methods, such as microarray [1]. The analytical methods for detecting hundreds to thousands of single nucleotide polymorphisms (SNPs) are very complex multistep procedures, which are liable to error [1]. Such errors could be translated into a risk misclassification, which in turn could make an individual feel a false sense of security or unnecessary anxiety [1]. So, analytical accuracy and credibility for risk estimation algorithms should be evaluated for commercialized genetic services. Although previous studies showed that the concordance of SNP calls between 23andMe and Navigenics was 99.7–100%, there were some considerable differences in the calculated relative risk of diseases in those studies [1, 2]. Newly developed Korean services have not been evaluated for SNP genotype accuracy and risk estimation of disease, yet. We aimed to compare Korean genetic services with 23andMe service for the evaluation.

## 2. Materials and Methods

**Figure 1:**
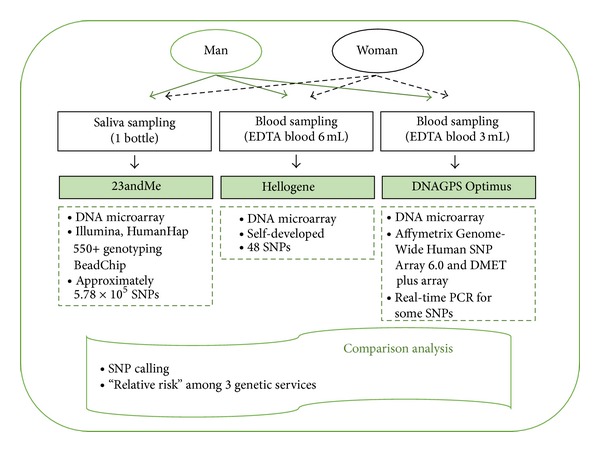
Schematic diagram of this study design.

This study was approved by the local institutional ethics committee (IRB number IB-3-1405-017). All subjects provided written informed consent. Schematic diagram of study design was shown in Figure 1. Samples were obtained from two volunteers, one healthy man (participant 1) and one healthy woman (participant 2). Samples were obtained with dedicated sample-collection kits according to each company's instruction. EDTA-anticoagulated blood samples were for Hellogene Platinum (Theragen Bio Institute, Suwon, South Korea) and DNAGPS Optimus (DNAlink, Seoul, South Korea), and saliva samples were for 23andMe (Mountain View, CA, USA).

Theragen Bio Institute used DNA microarrays SNP chip developed by themselves; DNAlink used DNA microarrays from Affymetrix GeneChip (Genome-Wide Human SNP Array 6.0 and DMET plus array) for the most of SNPs. Real-time PCR with TaqMan probe was used for some SNPs. 23andMe used DNA microarrays from Illumina with the HumanHap 550+ Genotyping BeadChip (approximately 5.78 × 10^5^ SNP) [1]. SNP call results and relative risks data for diseases were retrieved from formal customer reports of three services. The rs numbers of SNPs of DNAGPS Optimus and 23andMe were used for the evaluation of analytical SNP call concordance rate. But it was not available for Hellogene Platinum service because of proprietary concern of the manufacturer. The disease risk analysis of Korean services was known to be for the Korean population. For the 23andMe, we selected “Asian population” for the disease risk analysis through the 23andMe internet web page (https://www.23andme.com/).

## 3. Results

The numbers of SNPs used in each genetic service were shown in Table 1. All genetic services covered 16 disease categories. Mean 1.3 (range, 1-2) SNPs for each disease category were used in Hellogene Platinum service, while 3.7 (2–7) and 5.8 (1–15) SNPs were used in DNAGPS Optimus and in 23andMe service, respectively. Throughout 16 disease categories, 3 SNPs (rs9642880 for bladder cancer, rs1994090 for Parkinson's disease, and rs9939609 for obesity) were concurrently used in 3 services.

The comparison data of relative risks among 3 genetic services was shown in Table 2. The relative risks for several disease risk estimations were not concurred. For the lung cancer, its risk of participant 1 was very high in Hellogene Platinum and DNAGPS Optimus but was low in 23andMe. And the risk for participant 2 was high in Hellogene Platinum but was relatively low in DNAGPS Optimus and 23andMe.

The SNP calls concordance rate between services were done for DANGPS Optimus and 23andMe, 100% (30/30).

## 4. Discussion

This study was the first investigational study on Korean commercial genetic services for disease risk estimation. Each genetic service was based on an association between a specific genetic variant and a particular disorder [3]. The risk estimation was based on the data of several genome-wide association studies (GWAS), which showed the odds ratios between disease developing risk and genetic variants (SNP) in the huge population of an ethnic group. Most of the genetic variants tested for GWAS are weak predictors, accounting for only a small fraction of the overall heritability of a trait or disease, with the relative risk conferred being less than two [4, 5]. Being common, all individuals are likely to carry one or more risk alleles [6]. In our study, the highest relative risk was only 2.41 and mean relative risk was 1.02 ± 0.46. Even when GWAS demonstrated a strong association, there may not be any clinical utility [6]. One cannot base treatment decisions on such genome information, because most genotype information has no bearing on treatment strategy so far [6]. Most important concern is that commercial genetic services' predictive value must be sufficient to meet the standards for clinical use [3]. The clinical utility of a genetic test should be an essential criterion for deciding to offer this test to a person or a group of persons [3]. We emphasize that genetic services must be evaluated for clinical utility just as other medical procedures. In Korea, all newly introduced medical procedure and health technology should be applied to clinical field after being thoroughly evaluated by new health technology assessment (nHTA) system.

The results offered by commercial genetic services could inform health-related decisionssuch as lifestyle modification and medication [7]. Therefore analytical accuracy is essential. In this study, the concordance rate of SNP calls between DNAGPS Optimus and 23andMe services was 100% (30/30). This may be regarded as high concordance. The discrepant call for same SNP could gave totally oppositive interpretation. Continuous proficiency analysis and on-site inspection would be necessary for the improvement of analytical performance and user confidence.

Although methodology of GWAS has been developed through decades by standardization of study protocol and statistical method, it still has several issues and limitations. Lack of well-defined case and control groups, insufficient sample size, control for multiple testing, and control for population stratification are common problems [8]. Therefore, selection of optimal SNPs from qualified GWAS study is very important for calculation of individual disease risk. In this study, the numbers of SNPs for calculation for disease risk were different in each genetic service. While mean 1.3 (range, 1-2) SNPs for risk calculation were used in Hellogene Platinum, mean 3.7 (range, 2–7) SNPs and 5.8 (range, 1–15) SNPs were used for DNAGPS Optimus and 23andMe, respectively (Table 1). Also, the results of GWAS for the same disorder could be different according to the ethnic group; consequently the relative risks of genetic services entail different results. For example, the genotype of* SNCA *gene rs356220 CT means the relative risk of 1.02 for European but 0.96 for Asian (Participant 1). The selection of appropriate population for GWAS is very important. Some diseases listed in Korean genetic tests did not have even Asian genotype information, needless to say Korean data. The information obtained from Korean population should be more gathered into database.

In this study, each institute used their own calculation method for disease risk estimation. This was not only due to the lack of consensus guideline for selection of SNPs for risk calculation, but also due to use of unpublished data, which is produced by each institute. It could make selection bias, consequently making huge miscalculation of disease risk. For example, the SNP calls of rs2274223 were the same between DNAGPS Optimus and 23andMe services, but risks for esophageal cancer were different (1.85 for DNAGPS Optimus versus 1.21 for 23andMe for participant 1). Methodology of risk calculation must be validated and standardized.

For proper evaluation, each genetic service must provide the accurate and sufficient information, which is essential for objective and scientific interpretation and consultation, to doctors and patients. This information includes at least gene names, SNP names (rs number), used published references for risk calculation, test method, risk calculation method, and performance characteristics of the tests as other clinical genetic tests. If there is an interpretation guideline for specific genetic tests, each institute must follow this. In this study, even though there has been an established interpretation guideline for warfarin dosing (http://www.warfarindosing.org/) [9–11], DNAGPS Optimus does not interpret the results properly; consequently the dosing results of DNAGPS were opposite to those of 23andMe. What is worse is that Hellogene tested inapposite gene (*GGCX* gene) for warfarin dosing. This problem could be caused by lack of participation of medical specialist when these genetic services were developed. The genetic services offering the disease risk are not a merely genetic test for research but a medical test for clinical use. Therefore, doctors, especially laboratory physician, should be involved in the development and validation process of genetic services. In a case of other medical genetic tests, laboratory physician has responsibility of the whole test procedure and interpretative report, because its influences for patients are quite huge.

Laws and institution or clinical practices must be made ahead of consideration of commercialization to protect the privacy of the individual's genetic profile, which is the most private information [12]. There is some debate over a person's right to refuse to know that some genetic information has been engaged [12]. For these reasons, US Food & Drug Administration (FDA) ordered the 23andMe to stop marketing its health-related genetic test kit to consumers in November 2013 [12].

In conclusion, our study showed good concordance for SNP calls but discouraging concordance for disease risk estimation among three genetic services. Although they were performed by different companies using different assay platforms, we realized that these genetic services need standardization of interpretation algorithm and detailed evaluation of clinical utility.

## Figures and Tables

**Table 1:** The number of SNPs used for calculation of relative risk in three genetic services.

Category	The number of SNPs for all ethnic group (for Asian)			Number of overlapping SNPs among services	
	Hellogene Platinum	DNAGPS Optimus	23andMe		
Lung cancer	1 (1)	3 (2)	1 (0)	0	
Esophageal squamous cell cancer	2 (2)	5 (5)	1 (1)	2	(Hellogene and DNAGPS)
Colorectal caner	1 (1)	4 (3)	4 (2)	2	(DNAGPS and 23andMe)
Bladder cancer	1 (1)	3 (0)	6 (1) for male 7 (2) for female	1	(Three services)
Breast cancer (for female only)	1 (1)	5 (5)	8 (3)	1	(Hellogene and DNAGPS)
Atopic dermatitis	1 (1)	3 (1)	5 (1)	0	
Rheumatoid arthritis	1 (1)	4 (4)	9 (2)	1	(DNAGPS and 23andMe)
Systemic lupus erythematosus	1 (1)	3 (3)	6 (4)	0	
Psoriasis	2 (2)	4 (4)	3 (0)	1	(Hellogene and DNAGPS)
Diabetes mellitus, type 1	1 (1)	4 (0)	8 (0)	0	
Body mass index (obesity)	1 (1)	2 (2)	10 (0)	1 2	(Three services) (DNAGPS and 23andMe)
Atrial fibrillation	1 (1)	2 (0)	2 (0)	1	(Hellogene and DNAGPS)
Coronary artery disease	1 (1)	7 (4)	15 (0)	0	0
Parkinson's disease	2 (2)	4 (4)	10 (4)	1 1	(Three services) (Hellogene and 23andMe)
Narcolepsy	1 (1)	2 (1)	1 (0)	0	
Warfarin maintenance dose	1^†^	4^‡^	3^‡^	2	(DNAGPS and 23andMe)

^†^Warfarin dosing of Hellogene Platinum was based on *GGCX* gene genotyping results.

^‡^Warfarin dosing of DNAGPS Optimus and 23andMe was based on *CYP2C9 *and *VKORC1* gene genotyping results.

**Table 2:** Comparison of relative risks among 3 genetic tests.

Category	Relative risk for participant 1 (man)			Relative risk for participant 2 (woman)		
	Hellogene Platinum	DNAGPS Optimus	23andMe	Hellogene Platinum	DNAGPS Optimus	23andMe
Lung cancer	2.09	1.97	0.8∗	2.09	0.92	0.8∗
Esophageal squamous cell carcinoma	1.25	2.41	1.21	0.72	0.52	0.8
Colorectal caner	1.36	0.73	1.1	0.98	0.97	1.1
Bladder cancer	0.77	0.8	Typical	0.77	0.9	Typical/lower
Breast cancer	NA	NA	NA	0.84	0.77	0.98
Atopic dermatitis	1.16	1.13	Typical	1.16	0.74	Lower
Rheumatoid arthritis	NA	NA	0.71	0.97	2.95	1.1
Systemic lupus erythematosus	NA	NA	NA	0.97	1.47	1.09
Psoriasis	0.97	0.46	0.87∗	0.69	1.06	0.44∗
Diabetes mellitus, type 1	0.77	0.97	0.08∗	1.11	1.08	0.1∗
Body mass index (obesity)	0.91		0.85∗	0.91		0.83∗
Atrial fibrillation	0.94	0.68	1.12∗	0.94	1.39	1.93∗
Coronary artery disease	1.06	0.51	0.78∗	0.62	0.34	0.71∗
Parkinson's disease	1.09	0.84	1.05	0.95	1.04	0.77
Narcolepsy	1.41	1.29	Lower∗	1.41	1.29	Typical∗
Warfarin maintenance dose	Normal dose	Moderate dose	Decreased dose	Normal dose	Increased dose	Slightly decreased dose

NA: not applicable.

∗Relative risk in European. There was no Asian data.
